# Effects of blanching, acidification, or addition of EDTA on vitamin C and *β*‐carotene stability during mango purée preparation

**DOI:** 10.1002/fsn3.335

**Published:** 2016-01-18

**Authors:** Isabel R. F. Guiamba, Ulf Svanberg

**Affiliations:** ^1^ Departamento de Engenharia QuímicaFaculdade de EngenhariaUniversidade Eduardo MondlaneMaputoMozambique; ^2^ Departments of Biology and Biological Engineering/Food and Nutrition ScienceChalmers University of TechnologyGothenburgSweden

**Keywords:** Acidification, blanching, EDTA, mango, vitamin C, *β*‐carotene

## Abstract

The impact of acidification with citric acid, addition of EDTA or water blanching at high temperature, and short time (HTST) conducted at 90°C for 4 min, on the retention of vitamin C (L‐AA and DHAA) and *β*‐carotene was studied in mango purée 30 min after crushing. HTST blanching prior to matrix disruption into purée resulted in complete inactivation of polyphenol oxidase (PPO) and minor residual activity (8%) of ascorbic acid oxidase (AAO). The retention of total vitamin C was 100% in blanched purées and in purée with EDTA and about 90% in purées at pH 3.9 and 5.0. Acidification, blanching, and addition of EDTA preserved vitamin C mainly as L‐AA, while complete conversion into DHAA was observed in purée at pH 5.0. The retention of all‐*trans*‐*β*‐carotene was between 65 and 72%, with the highest value in purée with EDTA and the lowest value in purée of blanched mango. The ratio of 13‐*cis*‐*β*‐carotene in fresh mango was 8.2 ± 0.5% that increased significantly after blanching and in purée at pH 5.0.

## Introduction

Mango *(Mangifera indica* L*.)* is one of the most popular tropical fruits, due to its attractive yellow color and typical sweet aroma. Processed mango into juice or purée is commonly used as an intermediate product added to many different types of food systems, such as fruit juice blends, dairy products, and desserts. Mango is a rich source of dietary fiber and bioactive compounds such as provitamin A carotenoids, vitamin C, and phenolics (Pott et al. 2003; Rincon and Kerr 2010; Lemmens et al. 2013). Consumption of fruits and vegetables with such compounds are recommended for their health‐promoting effects in preventing several chronic diseases (Slavin and Lloyd 2012) and in providing provitamin A to populations susceptible to vitamin A deficiency (WHO 2009).

During the processes that involves tissue disruption, fruits and vegetables undergo progressive changes in quality due to enzymatic and nonenzymatic reactions, which decrease their physical and nutritional quality. Fresh‐cut or disrupted fruit and vegetable tissues have greater exposure to oxygen and oxidative enzymes, such as polyphenol oxidase (PPO) and ascorbic acid oxidase (AAO), thus promoting carotenoid oxidation, loss of vitamin C, and enzymatic browning (Rodriguez‐Amaya 1999; Spagna et al. 2005; Vásquez‐Caicedo et al. 2007; Munyaka et al. 2010a). To reduce the problems induced by enzymatic browning, thermal treatment, antibrowning, and chelating agents have been investigated. However, little information is found in the literature concerning the effect of antibrowning and chelating agents on nutrient retention.

The production of mango purée and juice may therefore involve several heating steps in order to inactivate oxidative enzymes and inhibit microbial growth. However, heat treatments may degrade carotenoids and vitamin C by nonenzymatic oxidation reactions that increase with tissue disruption (Vásquez‐Caicedo et al. 2007; Munyaka et al. 2010a; Lemmens et al. 2013). Inhibition of enzyme activity can also be achieved by reducing pH with carboxylic acids (Son et al. 2001; Arpita et al. 2010; Munyaka et al. 2010a) or by chelating Cu^2+^, a cofactor for AAO and PPO activity, with ethylenediaminetetraacetic acid (EDTA) (McEvily et al. 1992; Li 2014). It is thus generally recommended that mango purée should be acidified with citric acid to pH 4.0 or lower in order to prevent enzymatic browning and microbial spoilage (FAO 2001). A synergistic effect achieved by combining different antibrowning agents may result in an enhanced enzyme inhibition (Son et al. 2001).

Ascorbic acid oxidase is a copper‐containing enzyme that, in the presence of molecular oxygen, oxidizes ascorbic acid (L‐AA) to dehydroascorbic acid (DHAA), a highly unstable form that can rapidly undergo irreversible degradation to 2,3‐diketogulonic acid (DKGA) with a subsequent loss of vitamin C activity (Santos and Silva 2008). However, both L‐AA and DHAA exhibit vitamin C activity and it is therefore important to measure both L‐AA and DHAA in fruits and vegetables to find the true value of vitamin C (Oliveira et al. 2010).

Few studies on the effect of AAO during plant food processing are found in the literature (Yamaguchi et al. 2003; Munyaka et al. 2010b; Leong and Oey 2012; Guiamba et al. 2015). Munyaka et al. (2010b) studied the stability of vitamin C (L‐AA and DHAA) and AAO in crushed broccoli as influenced by different pretreatments. High temperature and short time (HTST) blanching or acidification prior to crushing resulted in a higher retention of vitamin C and a lower conversion of L‐AA to DHAA in the HTST‐blanched samples, thereby stabilizing vitamin C from further degradation of DHAA. Yamaguchi et al. (2003) investigated the influence of PPO and AAO on radical‐scavenging activity and contents of total phenols and ascorbic acid in several vegetables during cooking. The results showed that inactivation of both PPO and AAO by cooking retained the radical‐scavenging activity (in the form of total phenols and L‐AA) and may thereby indirectly prevent carotenoids from being degraded (Spagna et al. 2005).

The stability of *β*‐carotene during thermal processing of mango was studied by Vásquez‐Caicedo et al. (2007). A maximum loss of 7.2% for total *β*‐carotene was observed after pasteurization of purée at 90.5°C for 16 min. The PPO activity was completely inactivated after 1 min of holding time at the same temperature.

The aim of this work was to evaluate the influence of blanching, acidification, or an addition of a metal chelating agent, EDTA, on vitamin C and *β*‐carotene retention in the processing of mango purée. PPO and AAO were used as indicator enzymes affected by the blanching process.

## Materials and Methods

### Chemicals and standards

All chemicals for extraction and reagents were of analytical or HPLC grade obtained from Sigma–Aldrich (Stockholm, Sweden) or Fischer Scientific GTF (Göteborg, Sweden). The water was generated by Millipore Milli‐Q plus an ultrapure water system (Millipore, Solna, Sweden). All‐*trans*‐*β*‐carotene standard (synthetic, crystalline, Type II, product C‐4582), ACS grade L‐Ascorbic acid (≥99%), ethylenediaminetetraacetate disodium salt (EDTA), and Tris(2‐carboxyethyl) phosphine (TCEP) was obtained from Sigma–Aldrich (Stockholm, Sweden).

### Sample preparation

Two batches of mature, green‐ripe mango (*Mangifera indica* L.) of cv. ‘Keitt’ were purchased at a local market and stored for 3 days at 8–10°C. Then the fruits were randomly selected and kept for 24 h at room temperature before use. Bruised or wounded fruits were discarded and two to three mango fruits were used for each experiment. The fruits were washed with tap water, manually peeled and pitted. The fruits flesh was cut into small pieces, and used for the treatments described below, which were performed in duplicate. Fresh mango was used as a control for all the variables measured in all treatments. A portion of fresh mango for each treatment was immediately taken for vitamin C analysis, whereas samples for the enzymatic activity determinations and carotenoid analysis of fresh mango (50 g) were vacuum packed and stored at −80°C.

### Treatments

#### Unblanched mango purée at different pH

Fresh mango (100 g) was homogenized for 30 sec using a kitchen mixer (Braun MR 5550 MCA mixer, Germany) at two different pH levels of 0.2 mol/L citric acid buffer (pH 3.8 or pH 5.2 with or without 2 mmol/L EDTA) at a ratio of 1:1 (w/v). The final pH of the mango purées was 3.9 ± 0.1 or 5.0 ± 0.1. Samples for carotenoid analysis were immediately collected in vacuum‐packed plastic bags (OBH Nordic Food sealer, Sweden) and stored within 30 min at −80°C until analysis. Thirty minutes after crushing, portions of purées were taken for immediate vitamin C analysis.

#### Blanched mango purée

Two vacuum‐packed plastic bags of 100 g mango pieces (~1 cm thickness) each were subjected to high temperature and short time (HTST) blanching conducted at 90°C for 4 min followed by cooling in ice water for 5° min. Part of the blanched mango was immediately stored at −80°C until enzymatic activity determination, whereas the other part was crushed for 30 sec in the presence of 0.2 mol/L citric acid buffer (pH 5.2) at a ratio of 1:1 (w/v). A portion of purée was vacuum packed within 30 min and stored at −80°C for analysis of carotenoids, and the other portion was used for vitamin C extraction after 30 min.

#### Enzyme activity measurements

Enzymatic activities of fresh (control) and blanched mango were determined and the average of three measurements of the duplicates was recorded for polyphenol oxidase (PPO) and for ascorbic acid oxidase (AAO) enzymes.

### Polyphenol oxidase

#### Polyphenol oxidase extraction

Extraction and assay of PPO were carried out according to the procedure described by Palou et al. (1999) and followed by Ndiaye et al. (2009) with some modifications. Ten grams of fresh or blanched mango were mixed for 30 sec with 10 mL of McIlvaine citric‐phosphate buffer (pH 6.5) using a vortex mixer. The homogenates were transferred to 1 mL Eppendorf safe‐lock tubes and centrifuged at 13,400 g for 5 min in an IEC Micromax centrifuge (IEC – International Equipment Company, Needham Heights, MA, USA). The resulting clear supernatant was collected and stored on ice prior to enzyme activity measurements within the same day.

#### Polyphenol oxidase assay

The reaction solution consisted of 2 mL of McIlvaine citric‐phosphate buffer (pH 6.5), 1 mL of 0.175 mol/L 4‐methylcatechol solution, and 0.5 mL of the PPO extract. The assay mixture was incubated at 40°C for 20 sec before recording the absorbance at 420 nm for 3 min using a Cary 50 Bio UV‐visible spectrophotometer and semimicro polystyrene disposable cuvette (1.5 mL) with a light path of 10 mm. The blank consisted of 2.5 mL McIlvaine citric‐phosphate buffer solution (pH 6.5) and 1 mL of 4‐methylcatechol solution. Resulting data on the change in absorbance were plotted against time, and the PPO activity was calculated from the highest slope of the initial linear part of the curves for a period of 60 sec. The PPO activity (1 unit) was defined as an increase of absorbance of 0.001 at A_420_/min/g fresh weight. The residual activity of the enzyme was expressed as the percentage ratio of the slopes between blanched and corresponding fresh mango samples.

### Ascorbic acid oxidase

#### Ascorbic acid oxidase extraction

Ascorbic acid oxidase (AAO) activity was determined using the method described by Oberbacher and Vines (1963) and followed by Munyaka et al. (2010b) with some modifications. To extract AAO, 7.5 g of fresh or blanched mango was mixed with 15 mL of 0.1 mol/L phosphate buffer (pH 5.6, 0.5 mmol/L EDTA) and homogenized for 30 sec using a vortex mixer. The homogenates were transferred to 1 mL Eppendorf safe‐lock tubes and centrifuged at 13,400 g for 5 min in an IEC Micromax centrifuge. The resulting supernatant was stored on ice prior to enzyme activity measurements shortly thereafter.

#### Ascorbic acid oxidase assay

Ascorbic acid oxidase activity was determined by measuring the decrease in substrate concentration (0.5 mmol/L L‐AA) for 3 min using a Cary 50 Bio UV‐visible spectrophotometer at 25°C and 265 nm in a 70 *μ*L micro UV cuvette. The assay mixture consisted of 2.6 mL of 0.1 mol/L phosphate buffer (pH 5.6, 0.5 mmol/L EDTA), 100 *μ*L substrate, and 300 *μ*L enzyme extract. The blank consisted of 2.9 mL of 0.1 mol/L phosphate buffer (pH 5.6, 0.5 mmol/L EDTA) and 100 *μ*L of substrate solution. One unit of AAO activity was defined as a decrease in absorbance of 0.01 at A_265_/min/g fresh weight. The residual activity of AAO was expressed as the percentage ratio of the slopes between blanched and corresponding fresh mango samples.

### Determination of vitamin C (L‐AA and total ascorbic acid)

Vitamin C extraction and analysis were performed according to Munyaka et al. (2010a) with slight modifications. Approximately 0.3 g of fresh mango or 0.6 g of mango purée were homogenized using a vortex mixer in 15 mL of extraction buffer (20 mmol/L NaH_2_PO_4_, pH 2.1, 1 mmol/L EDTA). Tubes were allowed to stand for 5 min and then sonicated (Elmasonic S15, Elma, Singen, Germany) for 10 min and shaken well after 5 and 10 min. 1 mL of the homogenate was then centrifuged at 13,400 g for 5 min using an IEC Micromax centrifuge. Aliquots of the supernatant were diluted both in McIlvaine buffer alone (0.46 mol/L Na_2_HPO_4_, 0.27 mol/L citric acid, pH 4.5) and McIlvaine buffer containing 0.312 mmol/L TCEP (Tris [2‐carboxyethyl] phosphine hydrochloride). TCEP was used to reduce DHAA, thereby enabling the determination of both L‐ascorbic acid and total ascorbic acid. The samples with TCEP were incubated at room temperature for 60 min before HPLC analysis. All extractions were made in triplicate.

The analysis was performed using a HPLC system consisting of two pumps (Jasco PU‐2080Plus), a cooled auto sampler at 8°C (Jasco AS‐2057Plus) and an electrochemical detector (Decade II, Antec Leyden) equipped with a glassy carbon flow cell at 0.60 V (DC mode, range 50 nA). Separations were performed on a Thermo Aquasil C_18_ column (150 mm × 4.6 mm, particle size 3 *μ*m) and the mobile phase was phosphate buffer (50 mmol/L, pH 2.8) with a flow rate of 0.4 mL/min.10 *μ*L of filtered (0.45 *μ*m nylon filter) diluted extract was injected into the HPLC. L‐ascorbic acid standards were dissolved in McIlvaine buffer (0.46 mol/L Na_2_HPO_4_, 0.27 mol/L citric acid pH, 4.5, 0.312 mmol/L TCEP) in the range 1–50 *μ*g/mL and the calibration curve was used to quantify the L‐AA in the samples. One‐point calibration on each day of analysis was performed in order to verify detector response variability. Results of L‐AA and total vitamin C were expressed as milligrams per 100 g dry weight. The percentage retention of vitamin C was calculated as the ratio of vitamin C in the treated sample to the fresh sample × 100.

### Determination of *β*‐carotene (*trans/cis* isomers)

Carotenoid analysis was performed according to Bengtsson et al. (2008) with slight modifications. Approximately 0.2 g of finely ground flour of freeze dried fresh or processed mango was added to a test tube and vortexed after addition of 3.5 mL of ethanol:hexane mixture (4:3 by volume) containing 0.1% (w/v) butylated hydroxytoluene (BHT). The test tube was vortexed and then centrifuged at 4750 g for 5 min. The resulting supernatant was saved in a new test tube and the residue re‐extracted with 3.5 mL of ethanol:hexane mixture until the residue was colorless. To the resulting extract, 1.5 mL of petroleum ether was added together with 2 mL ultrapure water to aid in the separation of two phases. The organic and water phases were separated by centrifugation at 4750 g for 5 min, and the organic phase was transferred to a new tube. This step was repeated once. The pooled organic phase was evaporated in a heating block at 35°C under a stream of nitrogen. The residue was dissolved in 5 mL mobile phase (methanol:methyl tert‐butyl ether (7:3, v/v)).

Carotenoids were analyzed by reversed phase HPLC using a Waters 600 system equipped with an autosampler injector, degasser, pump and a Waters 996 UV–visible photodiode array detector operating at 450 nm. Absorption spectra were recorded between 250 nm and 500 nm. Separations were carried out on a C_30_ carotenoid column (5 *μ*m, 50 mm × 4.6 mm i.d.) (YMC Europe GMBH, Schermbeck, Germany). The injection volume was 20 *μ*L. Analytes were eluted from the column with differing proportions of methanol (MeOH) and methyl tert‐butyl ether (MTBE). The gradient initial conditions were 75% (v/v) MeOH and 25% (v/v) MTBE. At the 17th minutes, the ratio varied to 30% MeOH and 70% MTBE and at the 26th minutes to 75% and 25% MeOH and MTBE, respectively, up to finish at 30 min with a flow rate of 1 mL/min. The gradient allowed separations of all‐*trans*‐*β*‐carotene and its geometrical isomers (9, 13, and 15‐*cis*‐carotene). Quantification of all‐*trans*‐*β*‐carotene was done based on linear calibration curves of eight standard solutions of all‐*trans*‐*β*‐carotene and using response factors for quantification of the *cis* isomers according to Bengtsson et al. (2008). The concentration of all‐*trans*‐*β*‐carotene was expressed as micrograms per g dry matter, given as the mean of triplicate extractions. The percentage retention of all‐*trans*‐*β*‐carotene was calculated as the ratio of all‐*trans*‐*β*‐carotene in the treated sample to fresh sample × 100.

### Dry matter content

The dry matter (DM) content of fresh mango (%) was determined using a moisture balance incorporating the 310 M mass balance and HA 300 dryer (Precisa, Dietikon, Switzerland) with an IR‐heating device and an operating temperature of 70°C. The initial fresh sample weight was 5.0 ± 0.25 g (*n* = 3 for each purée preparation) and the sample was dried until constant weight.

### Statistical analysis

All the tests were performed in triplicate of duplicate samples and the results are presented as mean ± standard deviation. Differences between variables were tested for significance by one‐way analysis of variance (ANOVA) and Tukey′s HSD post hoc multiple range test. Differences were considered to be significant at *P* < 0.05 (or at a level of *α* = 0.05).

## Results and Discussion

The stability of vitamin C (L‐AA and DHAA) and carotenoids was investigated in mango purée subjected to acidification, addition of EDTA, or blanching at high temperature and short time (HTST) prior to homogenization. Green‐ripe mango flesh of the cv. ‘Keitt’ was crushed with added citric acid buffer to achieve a pH of 5.0 ± 0.1 with or without EDTA, representing the pH of ripe mango (Vazquez‐Salinas and Lakshminarayana 1985) and at pH 3.9 ± 0.1 that is within the recommended acidified pH for production of mango purée with improved microbial stability (FAO 2001). One portion of fresh mango pieces was subjected to HTST water blanching for a period of 4 min at 90°C in a closed plastic bag before crushing at pH 5.0, eliminating in this way the leaching of vitamin C. The nutrients (vitamin C and carotenoids) were analyzed in all puréed samples 30 min after crushing, which are referred to as crushed unblanched at pH 5.0 (pH5), crushed unblanched at pH 5.0 with EDTA (EDTA), crushed at pH 3.9 (pH4), and HTST blanched crushed at pH 5.0 (blanched).

### Thermal inactivation of PPO and AAO enzymes

Complete inactivation of PPO was achieved with the HTST thermal treatment of 4 min at 90°C, as no residual activity was detected in blanched mango samples. The PPO activity in the fresh mango was 121.6 ± 26.4 units, assuming no interference with the presence of vitamin C in the analysis as was shown by Ndiaye et al. (2009). High losses of PPO activity (98%) have been reported in mango pulp after blanching in 5 min at 80°C (Wang et al. 2007), and complete inactivation after 1 min of holding time at temperature levels between 85 and 93°C (Vásquez‐Caicedo et al. 2007) and after 2 min blanching at 90°C (Guiamba et al. 2015). The pH optimum for PPO of the mango cultivar Haden has been reported to be between 5.6 and 6.0 (Park et al. 1980), with only about 10% of the activity remaining at pH 4.

Minor AAO activity was detected after blanching with a retention of about 8% of the initial activity of 5.3 ± 0.25 units. Similar inhibition of AAO activity in mango of the Tommy Atkins cultivar (8% of the initial activity retained) was achieved after IR blanching at 90°C for 2 min (Guiamba et al. 2015). Cardello et al. (1993/1994) showed that AAO in the cellular wall of mango of the Haden cultivar was thermolabile above 80°C, whereas AAO in broccoli was completely inactivated at 80°C for 10 min (Munyaka et al. 2010a).

### Effects of acidification, addition of EDTA, and thermal treatment on the stability of vitamin C in mango purée

The average content of total vitamin C (L‐AA + DHAA) in fresh cv. *‘*Keitt’ mango varied from 73.9 to 121.0 mg/100 g dry weight (DW) (*n* = 8), corresponding to an average of 18.3 ± 4.1 mg/100 g fresh weight (FW). These values were based on the measured DM content of 20%. The total vitamin C content of fresh mango found in this study was within the range 11–134 mg/100 g FW reported by Manthey and Perkins‐Veazie (2009) in five mango cultivars, where cv. ‘Keitt’ had 25.6 ± 4.9 mg/100 g FW.

**Figure 1:**
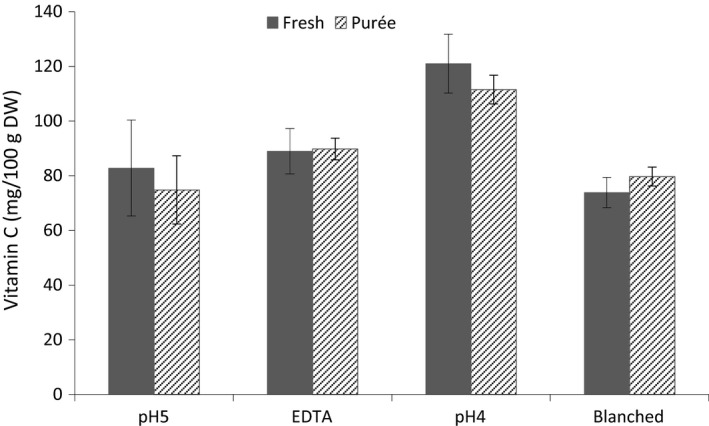
Total vitamin C (L‐AA and DHAA) content in fresh mango and mango purée 30 min after different treatments.

The retention of total vitamin C (L‐AA + DHAA) in the purée samples pH4 and pH5 was about 90%, compared with about 100% in purées of blanched mango and purées with added EDTA (Fig. 1). No significant differences were observed between fresh and processed samples, meaning that there were low losses of vitamin C as a result of irreversible DHAA degradation to 2,3‐diketogulonic acid (DKGA). In a study on thermal stability of L‐AA in raw crushed broccoli, Munyaka et al. (2010b) reported significant losses of vitamin C of about 20–40% after 15 min thermal treatments in the temperature range 30–90°C. HTST heating in water using a closed plastic bag that could avoid leaching of soluble solids was found to positively influence the total retention of vitamin C (L‐AA + DHAA) in this study. Significantly lower vitamin C retention (~60%) has recently been shown in HTST‐treated mango without plastic bag protection (Guiamba et al. 2015).

**Figure 2:**
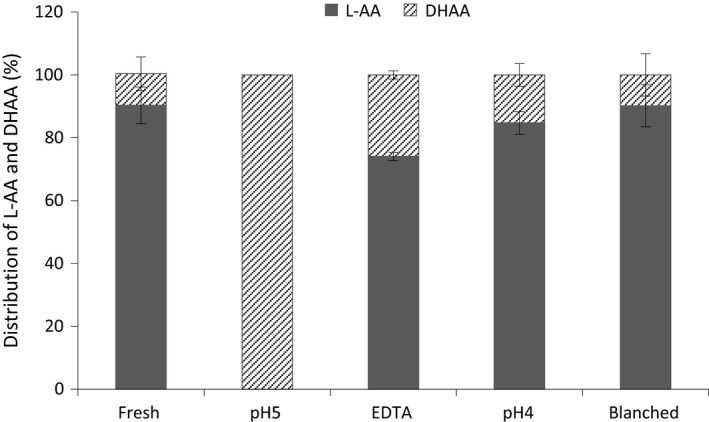
Percentage distribution of L‐AA and DHAA in fresh mango and mango purée.

Figure 2 shows the percentage distribution of L‐AA and DHAA in fresh mango and in puréed samples 30 min after being processed. The percentage distribution in fresh mango, and in purées of blanched and acidified (pH4) mango was similar, and vitamin C occurred mainly as L‐AA, 90.3 ± 5.9%, 90.2 ± 6.7%, and 84.7 ± 3.6%, respectively. However, the percentage distribution of L‐AA in EDTA purée was significantly lower, 74.0 ± 1.3%, while purée at pH 5.0 had a complete conversion into DHAA. Similar findings with a DHAA content of about 10% of the total vitamin C in fresh tropical fruits and vegetables including mango have been reported (Wills et al. 1984; Hernández et al. 2006; Gomez and Lajolo 2008) as well as in pasteurized (>80°C) carrots (Leong and Oey 2012). In two studies on broccoli, Munyaka et al. (2010a,b) reported both higher retention of vitamin C and higher ratio of L‐AA in blanched broccoli and puréed broccoli acidified with acetate buffer to pH 4.3

The results of this study can be explained by an almost complete inactivation of the AAO activity in purée of blanched mango, and a lowering of AAO activity as a result of low pH and chelating effect of EDTA. The complete conversion of L‐AA to DHAA in purée at pH 5.0 could be explained by an effective AAO activity at that pH. An optimal activity of AAO has also been reported at pH 5.5 for mango of the cultivar Haden (Cardello et al. 1993/1994). The acidification with citric acid to pH 3.9 may therefore be explained by the pH effect on the AAO activity rather than a chelating effect of citric acid on cupric ions as the main complexing capacity of citric acid is achieved at pH 6–7 (Warner and Weber 1953). Although acidification is a method commonly used for enzyme inhibition as well as for improved safety in fruit processing (FAO 2001), its application in fresh foods is restricted as it may adversely affect its flavor (Piližota and Šubaricć 1998).

The mango purée subjected to crushing with EDTA had a high percentage of L‐AA, which indicates that addition of EDTA during crushing could protect L‐AA from oxidative degradation to DHAA. The explanation may be that EDTA chelates the copper in the prosthetic group of the enzyme and thereby inhibits its activity. The results shows that the combination of citric acid with EDTA at pH 5.0 were more efficient in inhibiting the AAO activity than citric acid alone at pH 5.0, since complete oxidation of L‐AA to DHAA was observed with citric acid alone. It has been reported in the literature that combinations of inhibiting agents may result in enhancement of the inhibition (Son et al. 2001). Even if the retention of total vitamin C in this study was high in all processed mango purées, the high content of DHAA in purée at pH 5.0 may implicate further degradation of the vitamin C content during subsequent storage and/or processing. The results of this study therefore highlights the importance of taken into account the amount of DHAA in reporting vitamin C levels as recommended by Hernández et al. (2006).

### Effects of acidification, addition of EDTA, and thermal treatment on the stability of *β*‐carotene in mango purée

The amount of total *β*‐carotene in fresh cv. ‘Keitt’ mango was found to be between 8.2 and 10.7 mg/100 g dry weight (DW), which is an average of 1.9 ± 0.3 mg/100 g FW. This comprises 89.4% all‐*trans‐β*‐carotene and 10.6% 13‐*cis*‐*β*‐carotene, corresponding to 1.7 ± 0.2 mg/100 g FW and 0.2 ± 0.0 mg/100 g FW, respectively. Manthey and Perkins‐Veazie (2009) reported a *β*‐carotene content between 5 and 30 mg/kg among five mango cultivars, with the cv. ‘Keitt’ having 10.4 ± 2.4 mg/kg FW. Mercadante et al. (1997) found the amount of all‐*trans*‐*β*‐carotene in the same mango cultivar to be 1.5 ± 0.15 mg/100 g FW.

**Figure 3:**
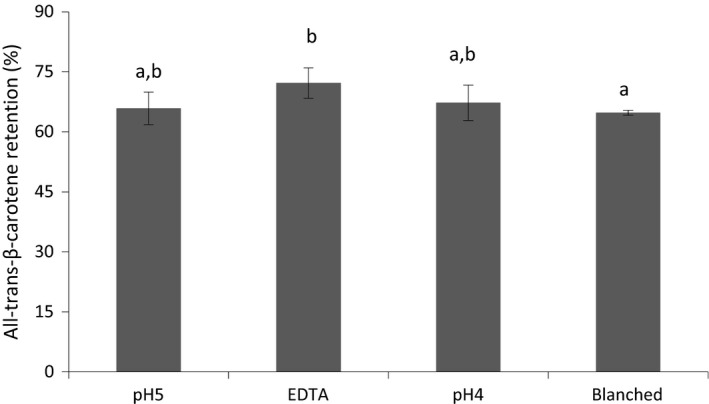
All‐*trans*‐*β*‐carotene retention in mango purée. Samples with different letters (a,b) are significantly different (*P* < 0.05).

The retentions of all‐*trans*‐*β*‐carotene in purées in relation to corresponding fresh mango are shown in Figure 3. In general, there was a significant degradation of all‐*trans*‐*β*‐carotene in all processed purées. The purée prepared with added EDTA had a significantly higher retention (72.2%) compared with blanched purée samples (64.8%). Crushing mango at pH 4 or 5 resulted in similar retentions of all‐*trans‐β*‐carotene, 67.2% and 65.9%, respectively. Any interactions with AAO and PPO activity thus seem unlikely. However, co‐oxidation of carotenoids as a result of PPO activity has been reported (Dorantes‐Alvarez and Chiralt 2000). On the other hand, Park et al. (1980) and Wang et al. (2007) noted that the PPO activity of mango pulp was negligible at pH 4.0. Our results on all‐*trans‐β*‐carotene retention in blanched mango purée are lower than those reported by Vásquez‐Caicedo et al. (2007). The explanation could be that Vásquez‐Caicedo and colleagues compared the pasteurized puréed samples with processed mango purée instead of with fresh mango as in our study. The purée processing step could have induced significant oxidative degradation of the all‐*trans‐β*‐carotene (Boon et al. 2010).

Polyphenol oxidase is a copper‐containing enzyme, which has been associated with the conversion of phenolic compounds to quinones and their products polymerization (Holzwarth et al. 2013). Therefore, EDTA can act as an inhibitor of PPO activity by chelating the copper in the prosthetic group of the enzyme (Sapers et al. 1989). Melo and Vilas Boas (2006) observed that EDTA at a level of 1% was the most efficient inhibitor of PPO activity in apple banana among combinations of different inhibitors. However, Luh and Phithakpol (1972) found only a slight decrease of peach PPO activity at an EDTA concentration of 0.26 mmol/L while, in our study, the final concentration of EDTA in the mango purée was 1 mmol/L.

The main changes that occur in carotenoids are due to the highly unsaturated structure of *β‐*carotene, which can be exposed to *trans‐cis*‐isomerization or oxidation, the last reaction being the major cause of carotenoid loss (Rodriguez‐Amaya 1999). The polyene chain is responsible for the light absorption properties and for the susceptibility of carotenoids to degradation by autooxidation, high temperature, and light (Boon et al. 2010). Disintegration of the food cell matrix caused by cutting and/or crushing can induce autooxidation and initial formation of *β*‐carotene isomers by the increased exposition to oxygen, and the process is stimulated by the presence of endogenous oxidative enzymes, metal ions, light, and co‐oxidation with lipid hydroperoxides (Rodriguez‐Amaya 1999; Boon et al. 2010).

Our findings that processing mango into purées resulted in some losses of all‐*trans*‐*β*‐carotene are consistent with the reports in the literature on mango (Vásquez‐Caicedo et al. 2007; Lemmens et al. 2013) and carrot purées (Lemmens et al. 2010). On the other hand, the higher amount of all‐*trans*‐*β*‐carotene in EDTA samples in comparison with blanched samples suggests that there might be some protective effect from EDTA. Qian et al. (2012) reported that EDTA was highly effective at inhibiting color loss (*β*‐carotene encapsulated within oil‐in‐water nanoemulsions), which was attributed to its ability to strongly chelate and inactive the transition metals (such as iron) that normally promote carotenoid oxidation.

**Figure 4:**
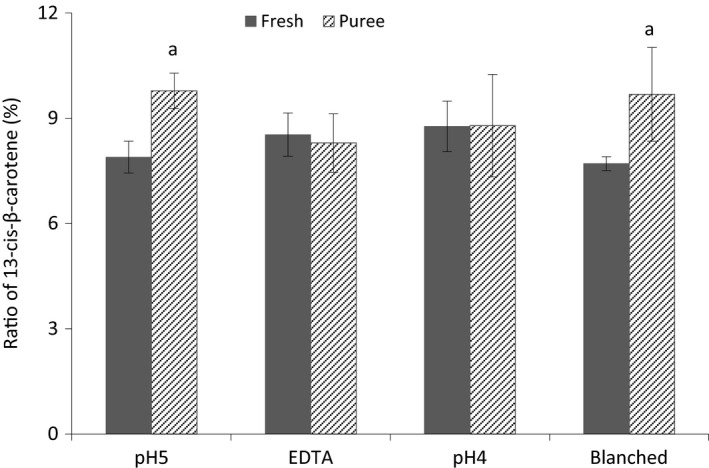
Percentage ratio of 13‐*cis*‐*β*‐carotene in relation to all‐*trans*‐*β*‐carotene in mango purée. Purée samples with a letter (a) are significantly different (*P* < 0.05) from the corresponding fresh mango.

The results on the ratio of 13‐*cis*‐*β*‐carotene (%) to all‐*trans*‐*β*‐carotene in fresh mango and in purée are presented in the Figure 4. No changes on the ratio of 13‐*cis*‐*β*‐carotene were observed in purées of EDTA and pH4, whereas crushing mango after blanching and at higher pH (pH5) induced *trans*‐*cis*‐isomerization of all‐*trans*‐*β*‐carotene, and as a result significantly higher ratio of 13‐*cis*‐*β*‐carotene was found. Similar results of increased *trans*‐*cis*‐isomerization of *β*‐carotene in mango purée at pasteurization temperatures of 85–93°C has been reported by Vásquez‐Caicedo et al. (2007). Severe heat treatment (>100°C) has also been shown to cause *β*‐carotene isomerization in carrot purée (Lemmens et al. 2010) and in mango purée (Lemmens et al. 2013). The retained 13‐*cis*‐*β*‐carotene ratio at pH4 and EDTA may be associated to reduced activity of PPO at pH 4, and inhibition of this enzyme by EDTA, respectively, as discussed above.

The combined results of a higher retention of all‐*trans*‐*β*‐carotene and retained ratio of 13‐*cis*‐*β*‐carotene in mango purées at pH 4 or in purées with added EDTA suggest an improved provitamin A activity of such purées.

## Conclusions

The results of this study show that the total retention of vitamin C was close to 100% in mango purée pretreated with a blanching step (90°C for 4 min) or puréed with addition of EDTA at a pH of 5.0, and about 90% in purées with added citric acid at pH 3.9 and 5.0, respectively. However, a complete conversion of L‐AA to DHAA was obtained in mango purée at pH 5.0, whereas vitamin C occurred mainly as L‐AA in fresh mango, and in purées of blanched and acidified (pH 3.9) mango (~90%) and in purée at pH 5.0 with EDTA (74%).

Although the retention of all‐*trans‐β*‐carotene was significantly reduced in all mango purées, the retention in mango purée with EDTA was significantly higher than in purée of blanched mango. No change in ratios of 13‐*cis*‐*β*‐carotene to all‐*trans‐β*‐carotene was observed in purées with EDTA and at pH 3.9, whereas the ratios increased in purées at pH 5.0 and in purées with an initial blanching step.

These results show that the disruption of the cellular matrix during purée processing facilitates oxidative reactions of vitamin C and of all‐*trans‐β*‐carotene unless protected by an initial blanching step, pH adjustments, or addition of a chelating agent EDTA to inhibit oxidative enzymes AAO and PPO.

## Conflict of Interest

The authors have no conflict of interest to declare and are responsible for the content and the writing of the manuscript.
